# To the Texas State Medical Association and the Medical Profession of Texas

**Published:** 1903-01

**Authors:** 


### TO THE TEXAS STATE MEDICAL ASSOCIATION AND THE MEDICAL PROFESSION OF TEXAS.

---

THE BILL FOR A STATE BOARD OF HEALTH, published herewith, was drawn up by the writer, as Chairman of the State Medical Association's Committee on Medical Legislation, after conference with State Health Officer Tabor—the head of the Quarantine Service. It was approved by him in all essentials, and at the request of the Chairman he was added to the Committee, and agreed to co-operate with them in securing the passage of the bill. The Chairman of the Committee accepted the appointment with the understanding that the bill should leave the Quarantine Department and the officers and laws of the same untouched; that it should not interfere with either, and upon this assurance from him, the State Health Officer consented to serve on the Committtee. The State Medical Association, at last session, having approved of the Harrison bill, which, it will be remembered, provided for this, and stipulated that the State Health Officer should be ex-officio President of of the Board (the bill was never introduced, for reasons given by Dr. Harrison, Chairman of Committee last year), I had assumed that there would be no objection to a similar provision in the present case. Indeed I thought it was generally understood by the Association and by the profession at large, that the above mentioned stipulations were the conditions precedent, the *sine qua non* —upon which we could hope to disarm the opposition of the Quarantine Department and its friends and defenders (which has, in every instance for the last score of years defeated every effort to secure the passage of a law creating a State Board of Health), and secure, instead, their co-operation. I had thought that it was well understood that it would not be again attempted to create a State Board of Health *de novo,* at the expense of the Quarantine Department, but to endeavor to *build upon* the existing health system, the skeleton of which exists but which in many essential details is inoperative because of defects in the law (a failure to provide penalties, etc., etc.); to make a State Board of Health and vital statistics *an annex* to the Quarantine Department. I deem it the part of wisdom, certainly of expediency, to ask for what there is a strong, or at least, a reasonable probability of our getting; and not for what is sure to be refused, as it has always been, in the past. The members of the legislature and the people at large are wedded to the State Quarantine and will not consent to its abolition or absorption. The quarantine officers are a unit in opposition to any measure that will interfere with it. They will unanimously, from the State Health Officer down to his humblest employe—and many, members of the Legislature from whom I have heard an expression, co-operate in the effort "to enlarge the scope and powers of the existing health system of the State for the better protection of the public health; to create and establish a State Board of Health and Vital Statistics *in conjunction with* the existing Quarantine Department; to create and put in operation county and municipal boards of health," etc.,—in brief, a provision for instituting measures of protection against diseases other than those now solely guarded against by quarantine. I thought that this was all understood and agreed to, and with that understanding I sought and obtained the support and co-operation of the quarantine men.

The bill was submitted to each member of the committee and greatly to my surprise—not to say chagrin—two of the members object to it; object to the very features that make it acceptable to the State Health Officer and his officers and friends. At this writing, January 6, two members of the committee have not been heard from. That cuts no figure, however, as the opposition of any one member spoils the undertaking and loses to the committee State Health Officer Tabor's co-operation and that of all of his officers, and the friends of the quarantine, as Dr. Tabor will withdraw. This lack of co-operation will have all the effect of active opposition, and the bill, if presented in other form will surely be defeated. This oppposition on the part of my colleagues places me in an embarrassing position in that it prevents my keeping faith with Dr. Tabor. I cannot make good the assurances I gave him,—and it renders me powerless to serve efficiently on the committee. I am a stayer, as all readers of the JOURNAL and all members of the Association know; but I am not a candidate for slaughter. I do not care to go up against certain defeat (I've been there twice) and go back to the Association in April and say again, *nihil fit.* There is nothing left for me to do, therefore, but withdraw from the committee, and let some gentleman who believes, in spite of the record of twenty years, that he can force the passage of a bill framed on other lines, have my place. I will give him the benefit of my labor in getting up this bill; will give him the bill. It was carefully drawn after much thought and consideration, and after a careful examination of the best laws in operation in other States. It is comprehensive and complete; it fills the requirements. It is adapted to the situation. While recognizing and accepting the existing status, it so dovetails in with the present system that it makes it complete and efficient. I am much disappointed at the opposition of my colleagues, and with a bow and thanks I turn the matter over to them.

I subjoin extracts from the report of the State Health Officer. It would be unreasonable to expect him to co-operate in securing any measure which would conflict with what he there recommends, and assigns him to a subordinate position, as proposed by the objecting members of the committee.

F. E. DANIEL, M. D.

(From report of State Health Officer, year ending August 31, 1902, pages 35, 36.

A STATE BOARD OF HEALTH.—"For many years efforts have been made by the committees apointed by the State Medical Association to have the Legislature authorize the creation of a State Board of Health. I think a Board of Health, organized in a proper manner and given authority to create health laws, would be of material benefit to the public. I favor strongly the creation of a State Board of Health, with the State Health Officer as ex-officio president of the board and its executive officer.

"I do not believe it advisable or expedient for the State Health Officer to be deprived of his present authority (which could be vested in him by a State Board of Health, nor do I believe the present system of coast and border quarantine should be interferred with.

"I am most heartily in favor of the creation of a bureau of vital statistics, which should be under the supervision of the head of this department."

---

THE AUSTIN DISTRICT MEDICAL SOCIETY held its regular annual meeting in Austin December 23rd, ult., and it was a rouser. Some of its members having accepted the hospitality of the "famous West Texas Medical Society," the A. D. reciprocated by inviting the W. T. to come to Austin to this meeting. Very few came, however, owing to a misunderstanding of the date and the near approach of Christmas time, but some of its representative members were present, as well as representatives from other societies, and we had a good time. An interesting program was carried out, some notably good papers having been presented and discussed with an enthusiasm that made one's soul feel good to hear. I recall an excellent paper on "Conservative Surgery," by Dr. Jos. Gilbert, of this place, surgeon to the Confederate Home; one by my late associate on the "Red Back," Dr. S. E. Hudson, reporting a case of congenital dislocation of the hip-joint and bloodless operation; one by Dr. H. B. Hill, of Austin, operation with success for reducing dislocation of the cervical vertebræ of four months' standing in a child of —— years; one by Dr. Joe S. Wooten, of Austin, on an operation (ligation of dorsal vein) for the cure of atonic impotence, with cases; one by Dr. Frank Paschal, of San Antonio, on suprà pelvic cystotomy for the relief of impervious stricture of the urethra. I do not intend to be invidious, but these are all the papers that I now recall that were read while I was present. They will all be published in the "Red Back" as soon as possible.

After labors, refreshments. The party, consisting of forty-five members and visitors, sat down to a square meal at the Café Milam at 7 p. m., and as they were to attend the performance of "The

Comedy of Errors" (Stuart Robson) at the opera house at 8, there was no time for a feast of wit and a flow of wine, nor, in fact, even beer. We had to cut it out—till after the performance. The theater boxes were all reserved for and occupied by the visiting doctors and their ladies. We feel that we have measurably evened up the score, and we Austin fellows can, with a clear conscience, again be the guests of the W. T. Oh, those San Antonio fellows know how to entertain hungry and thirsty meds.; they do it up brown. By the bye—they are to entertain the State Medical Association the fourth Tuesday in April,—just wait; something is going to happen then. The officers of the Austin District Medical Society for 1903 are President, Dr. F. E. Daniel, Austin; Vice-President, Dr. E. M. Thomas, Georgetown; Secretary and Treasurer, Dr. W. A. Harper, Austin.

---

THE BUBONIC PLAGUE is getting uncomfortably near us. It will be seen by the dispatches that it is prevailing with fearful mortality at Mazatlan, Mexico, and has made its appearance also in New Mexico and Arizona. We have a watchman on deck, however, who never sleeps; and what mortal man can do to keep it out of Texas—as he has kept out yellow fever—our vigilant and vigorous State Health Officer will do. If necessary he will not even let a bird from the infected places fly over Texas, nor cross the Rio Grande.

